# Acute Cannabinoids Produce Robust Anxiety-Like and Locomotor Effects in Mice, but Long-Term Consequences Are Age- and Sex-Dependent

**DOI:** 10.3389/fnbeh.2019.00032

**Published:** 2019-02-20

**Authors:** Chelsea R. Kasten, Yanping Zhang, Stephen L. Boehm

**Affiliations:** ^1^Department of Cell Biology and Anatomy, LSU Health Sciences Center New Orleans, New Orleans, LA, United States; ^2^Department of Psychology, Indiana University–Purdue University, Indianapolis, IN, United States; ^3^Indiana Alcohol Research Center, Indianapolis, IN, United States

**Keywords:** cannabinoids, THC, anxiety, cognition, sedation, mice, sex differences

## Abstract

The rise in cannabinoid legalization and decriminalization in the US has been paired with an increase in adolescents that perceive marijuana as a “no risk” drug. However, a comprehensive review of human literature indicates that cannabinoid usage may have both beneficial and detrimental effects, with adolescent exposure being a critical window for harming cognitive development. Although the cannabinoids Δ9-tetrahydrocannabinol (THC) and cannabidiol (CBD) are often used together for recreational and medical purposes, no study has previously observed the acute and long-lasting effects of THC+CBD in a battery of behavioral assays analogous to subjective human reports. The current study observed the acute and long-term effects of THC, CBD, and THC+CBD on object recognition memory, anxiety-like behavior, and activity levels in adolescent and adult mice of both sexes. Acute THC alone and in combination with CBD resulted in robust effects on anxiety-like and locomotor behavior. A history of repeated cannabinoid treatment followed by a period without drug administration resulted in minimal effects in these behavioral assays. Most notably, the strongest effects of repeated cannabinoid treatment were seen in adult females administered THC+CBD, which significantly impaired their object recognition. No effects of repeated cannabinoid history were present on hippocampal protein expression. These studies represent a detailed examination of age- and sex-effects of acute and repeated cannabinoid administration. However, the acute and long-term effects of THC with and without CBD on additional behaviors in adolescents and adults will need to be examined for a more complete picture of these drug effects.

## Introduction

Cannabinoids, such as Δ9-tetrahydrocannabinol (THC) and cannabidiol (CBD) found in marijuana (cannabis), bind to cannabinoid receptors (CBRs) and may disrupt well-maintained inhibitory signaling regulated by endogenous cannabinoids. Long-term effects of repeated use may persist even following a period of abstinence (Freund and Katona, 2007; Svizenksa et al., 2008; Chevaleyre and Piskorowski, 2014). Although cannabis usage rates have been relatively stable since 2002, the number of young adolescents and adults that report perceiving cannabis as a “no risk” drug has doubled to more than 17% in each age group (Azofeifa et al., 2016). However, no drug is fully without risks. A recent review by the National Academies of Sciences (2017) found that cannabinoid usage may have both beneficial and detrimental effects, but that adolescent exposure may be particularly harmful for cognitive development. This may be due to the increased expression and function of CB1Rs during early adolescence, which contribute to brain development (Romero et al., 1997; Verdurand et al., 2011; Lee and Gorzalka, 2012). Human research has demonstrated impairments in learning and memory even after cannabis use has ceased, with adolescent use linked to reduced educational and employment achievement.

Conclusions based on human research are weak and relatively constrained by methodological limitations (National Academies of Sciences, 2017). Several recommendations from the National Academies of Sciences include evaluating feelings of anxiety and sedation in all studies, focusing on the developmental period of adolescence, and including the use of preclinical studies examining both acute and chronic exposure to guide clinical research. Novel object recognition (NOR) is a preclinical analog of the human visual paired-comparisons task which harnesses a “normal” rodent’s preference for a novel object over a familiar one (Burbacher and Grant, 2012; Cohen and Stackman, 2015). Unconditioned anxiety-like activity may be assessed using the elevated plus maze (EPM) or quantifying the amount of total time spent in the center of an open field (Mohammad et al., 2016). Both of these measures compare an animal’s drive to remain in a “safe” space versus the drive to explore an open area, and open field activity also gives a measure of locomotor activity. NOR, EPM, and open field activity are optimal behavioral tasks for assessment of adolescent exposure, as they are quick, relatively free of stress, independent of external reward and punishment, and require minimal to no training (Cohen and Stackman, 2015; Mohammad et al., 2016). Our lab has previously demonstrated that mice can be selectively bred for resilience or susceptibility to the locomotor effects of THC, thereby highlighting the importance of monitoring this behavior (Kasten et al., 2018).

Using these tasks, preclinical studies have indicated both acute and long-term effects of cannabinoid exposure. Acute THC has been demonstrated to affect object recognition memory in CD-1 mice (Barbieri et al., 2016; Busquets-Garcia et al., 2018), but not in other rodent strains (Ciccocioppo et al., 2002; Long et al., 2010; Swartzwelder et al., 2012; Kasten et al., 2017). It reliably produces anxiogenic and sedative effects at higher doses (Onaivi et al., 1990; Célérier et al., 2006; Schramm-Sapyta et al., 2007; Lee et al., 2015; Kasten et al., 2017). A history of THC during adolescence results in memory impairments in the novel object task during adulthood in rats and mice (Quinn et al., 2008; Realini et al., 2011; Zamberletti et al., 2012; Kevin et al., 2017; Murphy et al., 2017, but see O’Tuathaigh et al., 2010; Cadoni et al., 2013; Segal-Gavish et al., 2017), but studies at the adult time-point have been inconclusive (Quinn et al., 2008; Vigano et al., 2009; Kasten et al., 2017; Rodríguez et al., 2017). Of particular interest, CBD is unable to independently alter NOR, but successfully rescues NOR deficits and proinflammatory responses in models of inflammation (Fagherazzi et al., 2012; Cadoni et al., 2013; Campos et al., 2015; Gomes et al., 2015). As adolescent THC administration results in a proinflammatory shift in the CNS during adulthood (Zamberletti et al., 2015), this may indicate that co-administration of CBD with THC may inhibit the NOR impairment demonstrated following adolescent treatment. Further, systemic and site-specific CBD exerts anxiolytic effects in the EPM via action at the serotonin 5HT1a receptor (Guimarães et al., 1990; Onaivi et al., 1990; Campos and Guimarães, 2008; Gomes et al., 2011; Marinho et al., 2015; Schiavon et al., 2016), and may thereby attenuate the anxiogenic effects of THC administration.

Although THC and CBD are often used together for recreational and medical purposes, no study has observed the acute and long-lasting effects of THC+CBD on the NOR, EPM, and open field tasks. Further, these assessments have not been systematically conducted in adolescents and adults of both sexes. The current work used adolescent (PND28) or adult (PND63) male and female C57Bl/6J (B6) mice. First, a dose-response to acute THC or CBD was assessed on EPM and open field activity to inform dose choices for repeated exposure. Following the dose-response studies, the acute effects of vehicle, 10 mg/kg THC, 20 mg/kg CBD, and THC+CBD were assessed for their effects on object recognition, EPM, and open field activity. Mice from the acute assessment received a total of eight injections over a 3-week period, then were given 3 weeks of rest. Following rest, all mice were again tested for object recognition, EPM, and open field activity under no-drug conditions to assess the effects of an adolescent or adult history of cannabinoids in male and female mice. Finally, protein levels of CB1R, interleukin 1 receptor 1 (IL-1R1), and serotonin 5HT1a receptors in the hippocampus were assessed. Although cannabinoids primarily work at cannabinoid receptors, CBD is known to exert behavior effects via the 5HT1a receptor (Russo et al., 2005; Campos and Guimarães, 2008). Further, repeated THC administration results in a pro-inflammatory shift in the CNS (Zamberletti et al., 2015), which may affect hippocampal-dependent memory due to changes in interleukin-1 signaling (Goshen et al., 2007). NOR tasks utilizing long delays are dependent on hippocampal function (Cohen and Stackman, 2015) and the hippocampus displays high levels of THC metabolites following acute administration (Leishman et al., 2018) making it a target region for these analyses.

## Materials and Methods

### Mice

A total of 440 male and female C57BL/6J (B6) mice were purchased from Jackson Laboratories and arrived at PND21 or PND56 and kept on a 12:12 h reverse light cycle. Mice were single-housed in Experiment 1 as in our previous work (Kasten et al., 2017). Although short-term social isolation during adolescence does not affect anxiety-like behavior in B6 mice, long-term isolation does (Lin et al., 2018). Further, object discrimination in the NOR task is not affected by social isolation alone, but is impaired when inflammatory processes are present in the hippocampus (Hueston et al., 2017). To avoid these potential confounds, mice were pair-housed in Experiments 2 and 3 due to the long-term nature of these experiments. All procedures adhered to the protocol approved by Indiana University-Purdue University Indianapolis School of Science Institutional Animal Care and Use Committee and conform to the Guidelines for the Care and Use of Laboratory Animals (Guide for the Care Use of Laboratory Animals, 2011) and the Public Health Service Policy on Human Care and Use of Laboratory Animals (National Institutes of Health, 2015).

### Drug

Both THC and CBD were generous gifts from the National Institutes of Health/National Institute on Drug Abuse (Bethesda, MD, United States). THC (1, 5, and 10 mg/kg), CBD (5, 10, and 20 mg/kg), or the combination (10 mg/kg THC + 20 mg/kg CBD) were dissolved in a vehicle solution of 5% Tween80, 5% 100 proof ethanol, and 90% saline. These doses cover the low to high ranges used throughout previous studies. Drug was administered in a pseudorandomized order, with each drug being equally represented in every cohort. Pair-housed mice received the same drug so that subordinate/dominate mates were equally represented across drug groups. All solutions were delivered via intraperitoneal injections in a volume of 0.1 mL per 10 g of body weight. To reduce the stress of multiple injections during Experiments 2 and 3 and mimic human use patterns, THC and CBD were combined in one solution. All injections were administered in the vivarium to keep the site of drug injection consistent.

### Behavioral Tasks

To maximize our ability to detect anxiogenic drug effects, injections and behavioral tasks were conducted during the active dark phase under red light conditions to potentially increase exploration time in the NOR task and amount of open arm time in the EPM. Because sex-related olfactory cues contribute to aggressive and territorial-related behaviors (Ervin et al., 2015), males and females were tested at different times and housed in different rooms to minimize exposure to the opposite sex.

#### Elevated Plus Maze

Mice were injected in the animal vivarium to avoid disruption of behavioral tasks by ultrasonic vocalizations used to communicate stressful and aversive stimuli, such as drug injections and restraint (Ko et al., 2005; Grimsley et al., 2016). Thirty minutes following injection, mice were individually transported approximately 30 feet to the EPM testing room (Kasten et al., 2017). Mice were placed in the EPM facing an open arm and given 5 min to explore. Two separate black Plexiglas plus mazes (Med Associates, Inc., St. Albans, VT, United States) adjusted for mouse size were used (see Moore et al., 2011 for details). Each session was video recorded and scored. Time in the open arms was recorded when all four of the animal’s paws crossed the center zone into the open arm. Each occurrence of four paws crossing into an open arm was counted as one open arm entry.

#### Open Field

Mice were individually transferred approximately 15 feet from the EPM room to the open field testing room. Each mouse was placed in a Versamax Animal Activity Monitor (Accuscan Instruments, Columbus, OH, United States) for 10 min without a habituation period. Locomotor activity was recorded by eight pairs of intersecting photocell beams (2 cm above the chamber floor) evenly spaced along the walls of the 40 × 40 cm test chamber. Sound-attenuating box chambers (inside dimensions, 53 cm across × 58 cm deep × 43 cm high) equipped with a house light and fan for ventilation and background noise encased the test chamber. The house light was off. The chambers were attached to a Dell computer which recorded activity counts every minute. Animals were immediately returned to their home cage in the vivarium following the session.

#### Novel Object Recognition

**FIGURE 1:**
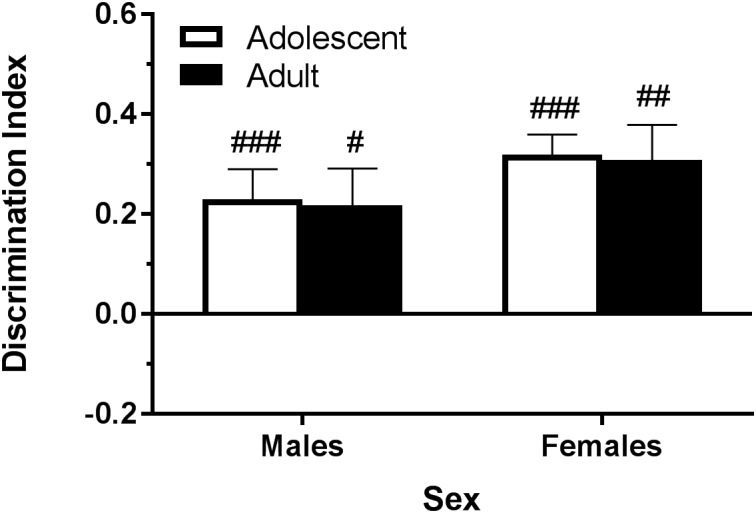
Depicts that, using the objects described in Table 1, pair-housed naïve B6 mice are able to significantly discriminate the novel object during the test phase. Pound sign indicates significantly different from zero at ^#^*p* < 0.05, ^##^*p* < 0.01, and ^###^*p* < 0.001, *n*’s = 8–10.

The NOR apparatus consists of a 40 × 40 × 40 cm wooden chamber painted light brown and sealed to block any spatial cues and allow for cleaning. The NOR task took place over 3 days, with each session being spaced 24 h apart. Sessions were recorded by a video camera and object investigation was hand-scored. On each day, the mice were individually walked into the testing room immediately prior to their session and returned to the vivarium immediately following their session. On the habituation day, animals were placed in the arena for 10 min without any objects present. On the training day, animals were placed into the arena with two identical objects and given 10 min to explore. The objects were placed approximately 10 cm out from diagonal corners. On the test day, one familiar object was replaced with a novel object in the test chamber, and mice were given 5 min to explore. Objects were optimized for each sex- and age-group in pair-housed naïve mice (Table 1 and Figure 1). Exploration time is time the animal spent oriented toward the object sniffing within 2 cm or in physical contact with the object.

**Table 1:** Indicates the objects used for the NOR task for each treatment and sex group at each time-point.

Treatment/Sex	PND28–30	PND70–72	PND111–113
Adolescent Males	Small “5 H Energy” and opaque drug vial	Small Erlenmeyer Pyrex and mini brown ceramic mug	
Adult Males		Small Erlenmeyer Pyrex and mini brown ceramic mug	Small “5 H Energy” and opaque drug vial
Adolescent Females	Small Erlenmeyer Pyrex and mini brown ceramic mug	Small conical tube and white plastic slide case	
Adult Females		Small conical tube and white plastic slide case	Small Erlenmeyer Pyrex and mini brown ceramic mug


### Experiments

#### Experiment 1: THC and CBD Dose Responses

Experiment 1 sought to characterize dose-response relationships to acute THC (0, 1, 5, or 10 mg/kg) and CBD (0, 5, 10, or 20 mg/kg) in adolescent (PND28-30) and adult (PND70+) B6 mice on anxiety-like and sedative behaviors. Mice received a 30-min cannabinoid pretreatment (Onaivi et al., 1990) before being placed on the EPM. Following the EPM task, mice were immediately transferred to and placed in the open field.

#### Experiment 2: Acute Effects of THC, CBD, and THC+CBD

Following the results Experiment 1, Experiment 2 assessed the effects of acute vehicle, 10 mg/kg THC, 20 mg/kg CBD, or THC+CBD on object recognition memory, anxiety-like behavior, and locomotor activity in adolescent (PND28) and adult (PND70) B6 mice. 10 mg/kg THC was chosen for its significant drug effects across all age^∗^sex groups, whereas 20 mg/kg CBD was chosen because it was the highest dose assessed in Aim 1. Although the ratio of THC:CBD varies greatly across both strains and laboratories, the 1:2 THC to CBD ratio is commercially available for recreational and medical use (Jikomes and Zoorob, 2018). For the NOR task, an acute injection was administered 10 min after conclusion of the training session and the test session occurred approximately 24 h later. A post-training injection time-point with a 24-h inter-trial interval has been used in previous studies observing the acute effects of THC on object recognition memory consolidation (Swartzwelder et al., 2012; Barbieri et al., 2016; Kasten et al., 2017; Busquets-Garcia et al., 2018) and avoids any cannabinoid-induced locomotor effects from interfering with object exploration. Acute effects of THC, CBD, or THC+CBD were also tested on EPM and open field. As in Experiment 1, mice were given a 30 min drug pretreatment before being placed on the EPM. Open field exploration was quantified immediately following testing on the EPM task.

#### Experiment 3: Aged Effects of THC, CBD, and THC+CBD

Experiment 3 assessed the effects of adolescent or adult cannabinoid history on later behavior. Mice from Experiment 2 received a total of eight injections of vehicle, 10 mg/kg THC, 20 mg/kg CBD, or THC+CBD over 3 weeks. These injections included the post-training NOR injection, the EPM pretreatment, and six additional maintenance injections. Mice then received 3 weeks of rest so that adolescent-treated mice could age to adulthood (PND70) and adult-treated mice aged to later adulthood (PND111). The NOR, EPM, and open field tasks were then run in the same manner as Experiment 2, with the exception that no drug was administered. See Table 2 for a timeline of Experiments 2 and 3.

**Table 2:** Details the timeline of Experiments 2 and 3 for each cohort.

	Monday	Tuesday	Wednesday	Thursday	Friday
Week 1		NOR habituation	NOR Training Injection 1	NOR Test	Injection 2 EPM and open field
Week 2	Injection 3		Injection 4		Injection 5
Week 3	Injection 6		Injection 7		Injection 8
Weeks 4–6	REST		
Week 7	NOR habituation	NOR Training	NOR Test	EPM and open field	Brain Extraction


### Hippocampal Western Blot

Approximately 24 h following completion of the aged behavioral tasks, mice were euthanized by cervical dislocation and brains were extracted (see Table 2). Whole brains were submerged in ice-cold autoclaved 1X PBS buffer for approximately 1 min. Brains were removed from the buffer, halved along the longitudinal fissure, and right and left whole hippocampi were removed. Samples were placed directly into 300 μl of ice-cold RIPA buffer with protease inhibitor (1 ml of RIPA buffer containing 100 μl of 10 × PI and 10 μl of 0.1 M PMSF) (Thermo Fisher) and frozen in a -80° F freezer. Details on tissue homogenization, sample denaturization, and Western Blot procedure can be found in Kasten et al. (2017). Western blots were run to identify levels of CB_1_R (Anti-Cannabinoid Receptor 1, Rabbit polyclonal, Abcam), interleukin 1 Receptor 1 (IL-1R1) (Anti-IL-1R1 antibody, Goat polyclonal, Thermo Fisher), and serotonin 5HT_1a_ receptor (Anti-5HT1a Receptor antibody, Rabbit polyclonal, Abcam). Protein expression for each mouse was calculated as the signal strength of protein of interest expression normalized to the signal strength of β-actin expression (Beta-actin Mouse Monoclonal Antibody, Li-Cor Biosciences Inc.).

### Statistical Analyses

All analyses were run separately in males and females to conserve statistical power to assess the primary question of these studies: does adolescent administration of cannabinoids differentially affect behavior compared to adult administration? Therefore, omnibus tests were Dose^∗^Age at Treatment for each sex independently. For all statistical analysis, the omnibus significance was set at *p* < 0.05 and corrected for follow-up tests. For Experiment 1, time in open arms, open arm entries, total locomotion in the open field, and percent of time spent in the center of the open field was analyzed using a Dose^∗^Age factorial ANOVA for THC and CBD. There was an *a priori* hypothesis that each age^∗^sex group may have different sensitivities to THC and CBD, so a one-way ANOVA analyzing dose response to each drug were run for all groups to determine dosage for Experiments 2 and 3. Dunnett’s *post hoc* tests were used to compare all drug doses to the vehicle group. Cohen’s *d* (*d*) is reported as a measure of effect size for all significantly different comparisons.

For Experiments 2 and 3, a Drug^∗^Age at Treatment factorial ANOVA was run to assess acute or prior history effects of vehicle, THC, CBD, or THC+CBD on novel object discrimination index, time in open arms, open arm entries, and activity in the open field for each sex independently. To reduce animal usage, n’s were kept at 8–10 and one-way ANOVAs corrected for statistical significance were run to assess the effect of dose or drug on each age group. Planned comparisons corrected for multiple analyses were used to analyze whether drug groups were significantly different from vehicle and whether THC+CBD was significantly different than THC alone for discrimination index, time in open arms, open arm entries, and open field activities. Pearson correlations within each age^∗^sex^∗^drug were used to determine whether investigation during the training session influenced discrimination index, as it has been previously suggested that more investigation during the training session may increase object recognition memory (Cohen and Stackman, 2015).

The discrimination index was calculated as (time spent with novel object – time spent with familiar object)/total object investigation time. It ranges from -1 to +1, with more positive numbers indicating more time spent with the novel object and 0 indicating no preference. Significant object discrimination is defined as a group being significantly different than 0 using a one-sample *t*-test. Percent of time spent in the center of the OF, an alternative measure of anxiety (Mohammad et al., 2016), were calculated as [(center activity/total activity)^∗^100]. This method of calculation controls for differences in total locomotion that may result from drug administration.

Western blots were analyzed using Drug^∗^Age at Treatment factorial ANOVAs, with follow-up one-way ANOVAs within each age group as described above. However, we also ran Sex^∗^Age at Treatment ANOVAs comparing the expression levels of the target protein in each vehicle group to discern whether there are any basal differences in expression levels.

## Results

### Experiment 1: THC and CBD Dose Responses

#### Elevated Plus Maze Activity

**FIGURE 2:**
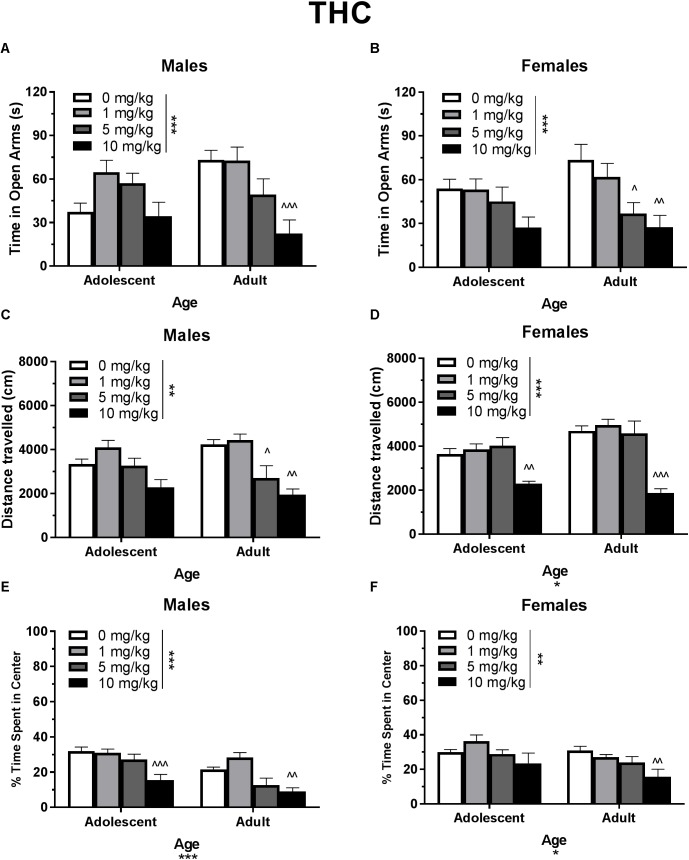
Depicts the effects of different doses of acute THC pretreatment in single-housed adolescent and adult mice on time in the open arms of the EPM (males, **A**; females **B**), total locomotion in the open field (males, **C**; females, **D**), and percent of time spent in the center of the open field (males, **E**; females, **F**). Asterisks indicate a significant main effect at ^∗^*p* < 0.05, ^∗∗^*p* < 0.01, and ^∗∗∗^*p* < 0.001. Carrots indicate significantly different from respective control at ^∧^*p* < 0.05, ^∧∧^*p* < 0.01, and ^∧∧∧^*p* < 0.001, *n*’s = 8–9.

In single-housed males, Dose^∗^Age ANOVAs revealed a significant interaction of THC on time spent in the open arms of the EPM [*F*(3,61) = 3.21, *p* < 0.05] (Figure 2A) as well as number of open arm entries [*F*(3,61) = 3.10, *p* < 0.05] (data not shown). There was not a significant effect of age on either metric, but there were significant dose effects [time in open arms *F*(3,61) = 7.71, *p* < 0.001; open arm entries *F*(3,61) = 6.76, *p* < 0.001]. One-way ANOVAs for each age group revealed that the 10 mg/kg dose reduced time spent in the open arms [*t*(29) = 4.13, *p* < 0.01, *d* = 2.12] and number of open arm entries [*t*(29) = 2.21, *p* < 0.01, *d* = 1.83] only in adult male mice. Adolescent open arm entries mean (*SEM*): Vehicle – 5.00 (*0.71*); 1 mg/kg THC – 8.88 (*1.14*); 5 mg/kg THC – 9.00 (*1.08*); 10 mg/kg THC 5.56 (*1.62*). Adult open arm entries mean (*SEM*): Vehicle – 9.88 (*1.30*); 1 mg/kg THC – 11.63 (*1.44*); 5 mg/kg THC – 8.00 (*1.80*); 10 mg/kg THC 3.33 (*1.16*).

**FIGURE 3:**
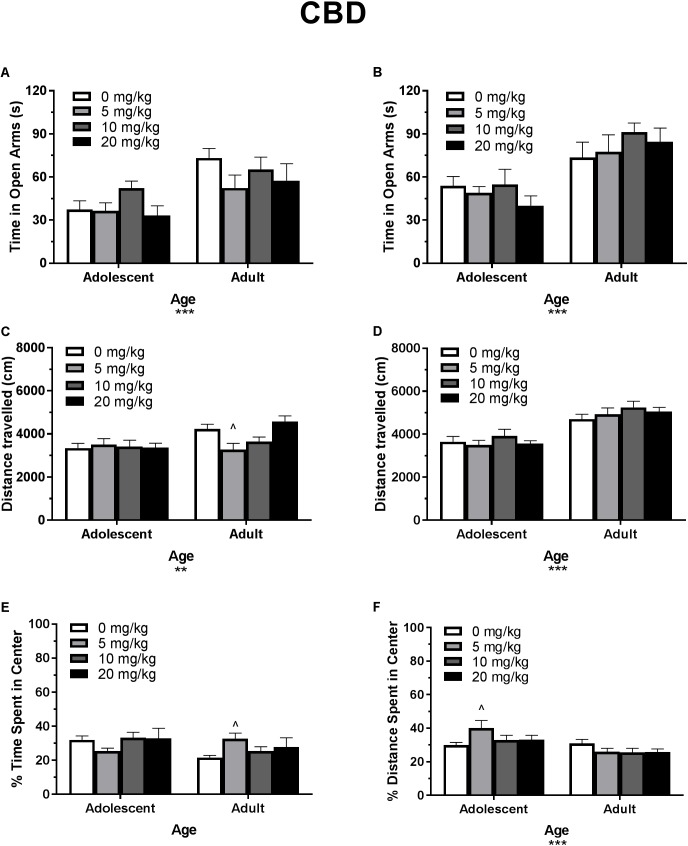
Depicts the effects of different doses of acute CBD pretreatment in single-housed adolescent and adult mice on time in the open arms of the EPM (males, **A**; females **B**), total locomotion in the open field (males, **C**; females, **D**), and percent of time spent in the center of the open field (males, **E**; females, **F**). Asterisks indicate a significant main effect ^∗∗^*p* < 0.01 and ^∗∗∗^*p* < 0.001. Carrot (^∧^) indicates significantly different from respective control at *p* < 0.05, *n*’s 7–9.

In single-housed males, Dose^∗^Age ANOVAs revealed no interaction or main effect of dose of CBD on time in the open arms (Figure 3A) or number of open arm entries in males (*p*’s > 0.05) (data not shown). There was a significant effect of age for both variables, with adults spending more time in the open arms [*F*(1,56) = 17.13, *p* < 0.001] and making more open arm entries compared to adolescents [*F*(1,56) = 16.31, *p* < 0.001]. Adolescent open arm entries mean (*SEM*): Vehicle – 5.00 (*0.71*); 5 mg/kg CBD – 5.50 (*0.80*); 10 mg/kg CBD – 6.25 (*1.00*); 20 mg/kg CBD 5.22 (*0.94*). Adult open arm entries mean (*SEM*): Vehicle – 9.88 (*1.30*); 5 mg/kg CBD – 7.29 (*1.06*); 10 mg/kg CBD – 8.00 (*1.00*); 20 mg/kg CBD 5.22 (*0.94*).

In single-housed females, Dose^∗^Age ANOVAs revealed no significant interaction or effect of age on time in the open arms (Figure 2B) or number of open arm entries following THC administration (*p*’s > 0.05) (data not shown). However, there was a significant effect of THC dose on both variables [time in open arms *F*(3,60) = 8.08, *p* < 0.001; open arm entries *F*(3,60) = 3.35, *p* < 0.05]. In adult mice, the 5 and 10 mg/kg doses of THC reduced time spent in the open arms [5 mg/kg *t*(29) = 3.53, *p* < 0.05, *d* = 1.40; 10 mg/kg *t*(29) = 3.69, *p* < 0.01, *d* = 1.68] as well as number of open arm entries [10 mg/kg *t*(29) = 3.06, *p* < 0.05, *d* = 1.43]. There were no significant dose effects of THC in adolescents (*p* > 0.05). Adolescent open arm entries mean (*SEM*): Vehicle – 7.44 (*0.74*); 1 mg/kg THC – 6.22 (*0.64*); 5 mg/kg THC – 7.25 (*1.58*); 10 mg/kg THC 5.11 (*1.25*). Adult open arm entries mean (*SEM*): Vehicle – 9.88 (*1.22*); 1 mg/kg THC – 9.25 (*1.74*); 5 mg/kg THC – 6.75 (*1.37*); 10 mg/kg THC 1.22 (*1.22*).

In single-housed females, Dose^∗^Age ANOVAs revealed no interaction or main effect of dose of CBD on time in the open arms (Figure 3B) or number of open arm entries in females (*p*’s > 0.05) (data not shown). There was a significant effect of age for both variables, with adults spending more time in the open arms [*F*(1,59) = 27.75, *p* < 0.001] and making more open arm entries compared to adolescents [*F*(1,59) = 27.62, *p* < 0.001]. Adolescent open arm entries mean (*SEM*): Vehicle – 7.44 (*0.74*); 5 mg/kg CBD – 6.5 (*1.00*); 10 mg/kg CBD – 7 (*1.05*); 20 mg/kg CBD 5.44 (*0.80*). Adult open arm entries mean (*SEM*): Vehicle – 9.88 (*1.22*); 5 mg/kg CBD – 9.38 (*1.28*); 10 mg/kg CBD – 12.63 (*1.02*); 20 mg/kg CBD 4.70 (*1.57*).

#### Total Locomotion in the Open Field

In single-housed males, a Dose^∗^Age ANOVA revealed no significant interaction or main effect of age on THC-induced locomotor activity in the open field (*p*’s > 0.05) (Figure 2C). There was a significant main effect of dose [*F*(3,62) = 15.50, *p* < 0.001]. One-way ANOVAs for each age revealed that the 5 and 10 mg/kg doses reduced total locomotion, but only in adult mice [5 mg/kg *t*(31) = 3.07, *p* < 0.05, *d* = 1.21; 10 mg/kg *t*(31) = 4.59, *p* < 0.001, *d* = 3.19)]. Reduced activity in 5 mg/kg adult group was significantly correlated with reduced time in the open arms [*r*(9) = 0.682, *p* < 0.05] and percent of time spent in the center of the open field [*r*(9) = 0.886, *p* < 0.01]. Reduced activity in the 10 mg/kg adult group was not significantly correlated with anxiety-like metrics (*p*’s > 0.05). For CBD, a Dose^∗^Age ANOVA revealed a significant interaction [*F*(3,61) = 3.42, *p* < 0.05] and main effect of age [*F*(1,61) = 9.20, *p* < 0.01] (Figure 3C), with adults moving more. Although there was no main effect of dose, one-way ANOVAs for each age group revealed that the 5 mg/kg dose reduced total locomotion in adults [*t*(31) = 2.67, *p* < 0.05, *d* = 1.31]. Reduced activity in this group was not significantly correlated with anxiety-like metrics (*p*’s > 0.05).

In single-housed females, a Dose^∗^Age ANOVA revealed no significant interaction on THC-induced locomotor activity in the open field (*p* > 0.05) (Figure 2D). There were significant effects of age [*F*(1,61) = 6.95, *p* < 0.05] and dose [*F*(3,61) = 26.85, *p* < 0.001]. Overall, adults moved more, and the 10 mg/kg dose reduced activity in both age groups [(adolescent *t*(31) = 3.86, *p* < 0.01, *d* = 2.38; adult *t*(30) = 5.78, *p* < 0.001, *d* = 4.46]. Reduced activity was not significantly correlated with anxiety-like metrics in either age group (*p*’s > 0.05). For CBD, a Dose^∗^Age ANOVA revealed only a significant effect of age on total locomotion, with adults moving more [*F*(1,59) = 59.23, *p* < 0.001] (Figure 3D). One-way ANOVAs for each age group also revealed no significant effects of CBD dose on total locomotion.

#### Percent of Time Spent in the Center of the Open Field

In single-housed males, a Dose^∗^Age ANOVA revealed no significant interaction of THC on anxiety-like activity in the open field (*p* > 0.05) (Figure 2E). There was a significant main effect of age [*F*(1,62) = 19.77, *p* < 0.001], with adults spending significantly less time in the center of the open field as a percent of overall time moving, indicating a more-anxious phenotype than adolescents. There was also a significant anxiogenic effect of THC dose [*F*(3,62) = 16.27, *p* < 0.001], with 10 mg/kg decreasing the percent of time spent in the center of the open field [adolescent *t*(31) = 4.33, *p* < 0.001, *d* = 1.95; adult *t*(31) = 3.36, *p* < 0.01, *d* = 2.43]. For CBD, a Dose^∗^Age ANOVA revealed no significant interaction or main effect of dose or age on anxiety-like activity (*p*’s > 0.05) (Figure 3E). One-way ANOVAs at each age revealed that 5 mg/kg CBD significantly increased percent of time spent in the center of the open field in adult males [*t*(31) = 2.72, *p* < 0.05, *d* = 1.88].

In single-housed females, a Dose^∗^Age ANOVA revealed no significant interaction of THC on anxiety-like activity in the open field (*p* > 0.05) (Figure 2F). There was a significant main effect of age [*F*(1,61) = 4.20, *p* < 0.05], with adults spending significantly less time in the center of the open field as a percent of overall time moving, indicating a more-anxious phenotype than adolescents. There was a significant main effect of dose [*F*(3,61) = 4.58, *p* < 0.01] with 10 mg/kg significantly decreasing the percent of time spent in the center of the open field only in adults [*t*(30) = 3.35, *p* < 0.01, *d* = 1.43]. For CBD, a Dose^∗^Age ANOVA revealed a significant interaction on percent of time spent in the center of the open field [*F*(3,59) = 2.88, *p* < 0.05]. There was no main effect of dose (*p* > 0.05), but there was a significant main effect of age [*F*(1,59) = 14.02, *p* < 0.001], with adults demonstrating a more-anxious phenotype than adolescents (Figure 3F). One-way ANOVAs at each age revealed that 5 mg/kg CBD significantly increased percent of time spent in the center of the open field in adolescent females [*t*(29) = 2.43, *p* < 0.05, *d* = 1.07].

### Experiment 2: Acute Effects of THC, CBD, and THC+CBD

#### Novel Object Recognition

**FIGURE 4:**
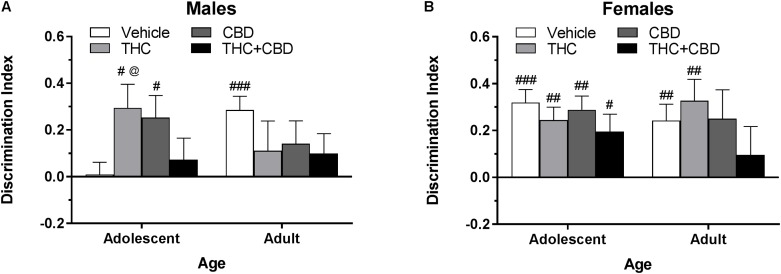
Depicts discrimination of a novel object in the NOR task in pair-housed adolescent and adult males **(A)** and females **(B)** when cannabinoids were administered post-training. Higher positive values indicate more time spent with the novel object. Hashtag indicates significant discrimination (different from 0) at ^#^*p* < 0.05, ^##^*p* < 0.01, and ^###^*p* < 0.001. Ampersand (@) indicates a trend toward different from control at *p* = 0.05 – 0.075, *n*’s = 9–10.

In pair-housed males, one-sample *t*-tests indicated that significant novel object discrimination occurred in adolescents treated with THC and CBD and adults treated with vehicle following object training (*p*’s < 0.05) (Figure 4A). A Drug^∗^Age ANOVA revealed no significant effects of drug or age (*p*’s > 0.05), but a trend toward an interaction [*F*(3,71) = 2.47, *p* = 0.069]. One-way ANOVAs examining drug effects within each age group indicated a trend toward a significant effect in adolescents [*F*(3,35) = 2.51, *p* = 0.074] with THC trending toward increasing object discrimination compared to vehicle [*t*(35) = 2.30, *p* = 0.071, *d* = 1.17]. There were no significant effects of drug in the adult groups (*p* > 0.05). Importantly, the lack of differences in total object investigation time during the training and test sessions (data not shown) indicate that these differences in discrimination index are not due to basal or drug-induced motivational differences in investigation. Discrimination index was not significantly correlated with training investigation time within any drug group (*p*’s > 0.05) (data not shown).

In pair-housed females, one-sample t-tests indicated that significant novel object discrimination occurred in all adolescent-treated groups, as well as in adults treated with vehicle and THC following object training (Figure 4B). There were no significant main effects of age or drug, nor interaction of the two variables, on discrimination index (*p*’s > 0.05). Further, there were no significant drug effects within each age group (*p*’s > 0.05). While all groups spent a similar amount of time investigating the objects during training, acute CBD increased object investigation during the test phase in adolescent females [*t*(36) = 3.10, *p* < 0.05] (data not shown). Discrimination index was significantly positively correlated with training investigation in adolescents treated with vehicle [*r*(10) = 0.701] and adults treated with THC+CBD [*r*(10) = 0.683] (*p*’s < 0.05) (data not shown).

#### Elevated Plus Maze Activity

**FIGURE 5:**
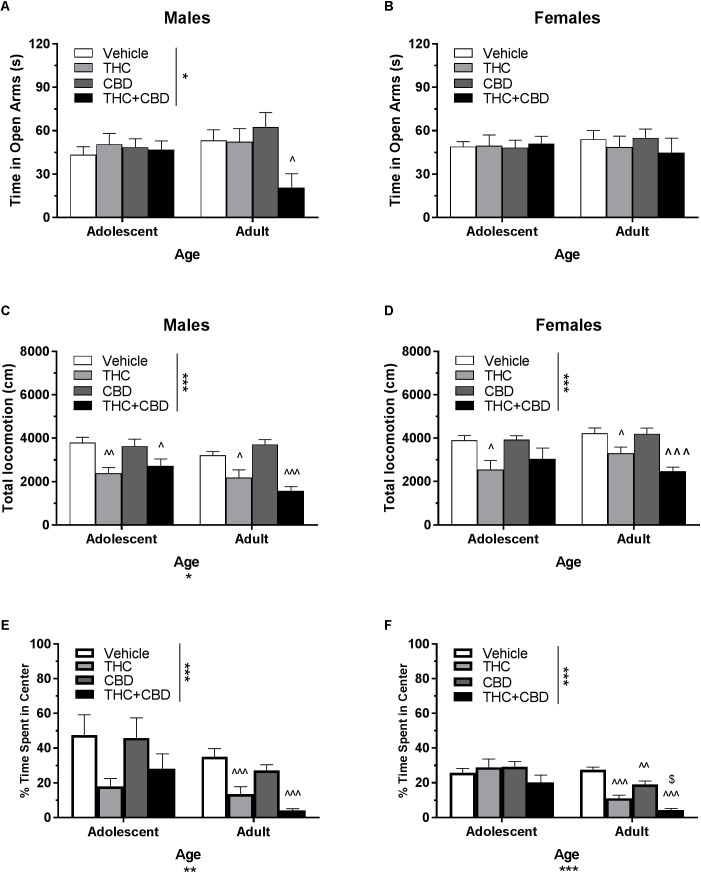
Depicts the effects of acute cannabinoid pretreatment in pair-housed adolescent and adult mice on time in the open arms of the EPM (males, **A**; females **B**), total locomotion in the open field (males, **C**; females, **D**), and percent of time spent in the center of the open field (males, **E**; females, **F**). Asterisks indicate a significant main effect at ^∗^*p* < 0.05, ^∗∗^*p* < 0.01, and ^∗∗∗^*p* < 0.001. Carrots indicate significantly different from respective control at ^∧^*p* < 0.05, ^∧∧^*p* < 0.01, and ^∧∧∧^*p* < 0.001. Dollar sign ($) indicates significantly different from respective THC group at *p* < 0.05, *n*’s = 7–10.

In pair-housed males, a Drug^∗^Age ANOVA revealed a strong trend toward an interaction of drug treatment and age on time spent in the open arms of the EPM following acute cannabinoid pretreatment [*F*(3,67) = 2.73, *p* = 0.0505]. There was no significant effect of age (*p* > 0.05), but there was a main effect of drug (*p* < 0.05). One-way ANOVAs for each age group indicated a significant effect of drug in adults [*F*(3,32) = 4.13, *p* < 0.05], with THC+CBD reducing time in the open arms [*t*(32) = 2.61, *p* < 0.05, *d* = 1.26) (Figure 5A). For open arm entries, a Dose^∗^Age ANOVA also revealed a significant interaction [*F*(3,67) = 2.86, *p* < 0.05] and effect of dose [*F*(3,67) = 2.91, *p* < 0.05], but no significant effect of age (*p* > 0.05). One-way ANOVAs for each age group revealed that THC significantly increased open arm entries in adolescent males compared to vehicle [*t*(35) = 2.60, *p* < 0.05, *d* = 0.64], but that no adult group was significantly different from control (*p* > 0.05) (data not shown).

In pair-housed females, analyses revealed no significant interaction of Dose^∗^Age, no significant effect of age, and no effects of drug on time spent in the open arms (Figure 5B) or number of open arm entries (*p*’s > 0.05) (data not shown).

#### Open Field Activity

In pair-housed males, a Drug^∗^Age ANOVA revealed no significant interaction on total locomotion in the open field (*p* > 0.05). There was a significant effect of age [*F*(1,72) = 5.91, *p* < 0.05], with adolescents traveling a greater distance overall. There was also a significant effect of drug [*F*(3,72) = 17.46, *p* < 0.001], with THC and THC+CBD significantly reducing total locomotion in both age groups [adolescent THC *t*(36) = 3.45, *p* < 0.01, *d* = 1.76; adolescent THC+CBD *t*(36) = 2.64, *p* < 0.05, *d* = 1.19; adult THC *t*(36) = 2.91, *p* < 0.05, *d* = 1.15; adult THC+CBD *t*(36) = 4.64, *p* < 0.001, *d* = 2.75] (Figure 5C). In all groups with the exception of adults administered THC+CBD, total locomotion was significantly positive correlated with percent time in the center of the open field (*p*’s < 0.01), but not significantly correlated with time spent in the EPM open arms (*p*’s > 0.05). Anxiety-like activity in the open field was quantified using percent of time spent in the center of the open field. A Drug^∗^Age ANOVA revealed no significant interaction (*p* > 0.05), but a significant effect of age [*F*(1,72) = 8.52, *p* < 0.01] and effect of drug [*F*(3,72) = 6.86, *p* < 0.001] on anxiety-like activity following acute cannabinoid administration. Adolescents demonstrated increased percentage of time spent in the center of the open field compared to their adult counterparts, indicating a less-anxious phenotype. One-way ANOVAs for each age indicated no significant effect of cannabinoid pretreatment in adolescents (*p* > 0.05), but that THC and THC+CBD significantly decreased the percentage of total locomotion in the center of the open field in adults, indicating anxiogenic effects [THC *t*(36) = 4.2, *p* < 0.001, *d* = 1.51; THC+CBD t(36) = 6.03, *p* < 0.001, *d* = 2.85] (Figure 5E).

In pair-housed females, a Drug^∗^Age ANOVA revealed no significant interaction or main effect of age on total locomotion in the open field (*p*’s > 0.05). There was a significant main effect of drug [*F*(3,71) = 10.43, *p* < 0.001], with THC reducing total locomotion in both age groups [adolescent *t*(36) = 2.68, *p* < 0.05, *d* = 1.26; adult *t*(35) = 2.64, *p* < 0.05, *d* = 1.08], and THC+CBD reducing total locomotion only in adults [*t*(35) = 4.85, *p* < 0.001, *d* = 2.61] (Figure 5D). Reduced activity was not correlated with anxiety-like activity in the open field and EPM (*p*’s > 0.05). A Drug^∗^Age ANOVA revealed a significant interaction [*F*(3,71) = 4.57, *p* < 0.01], effect of age [*F*(1,71) = 26.12, *p* < 0.001], and effect of drug [*F*(3,71) = 9.12, *p* < 0.001] on anxiety-like activity following acute cannabinoid administration. As with males, female adolescents demonstrated increased percentage of time spent in the center of the open field compared to their adult counterparts, indicating a less-anxious phenotype. Cannabinoid pretreatment did not significantly alter this behavior in adolescent females (*p* > 0.05). In adults, all cannabinoids significantly reduced percentage of time spent in the center [THC *t*(35) = 7.10, *p* < 0.001, *d* = 3.11; CBD *t*(35) = 3.68, *p* < 0.01, *d* = 1.48; THC+CBD *t*(35) = 9.78, *p* < 0.001, *d* = 5.81], and THC+CBD decreased this parameter synergistically when compared to THC-alone [*t*(35) = 2.87, *p* < 0.05, *d* = 1.55] (Figure 5F).

### Experiment 3: Aged Effects of THC, CBD, and THC+CBD

#### Weight

One-way ANOVAs on weight at the first drug administration, the last drug administration, and the aged testing point indicated no significant differences in baseline weight, weight gain over injections, or long-term weight gain in any group (*p*’s > 0.05, Table 3).

**Table 3:** Displays the mean weight ± standard error for the first day of drug treatment, the last day of drug treatment, and the aged behavior testing point for all mice in Aims 2 and 3.

	Weight (g ± SEM)		
	
	Vehicle	THC	CBD	THC+CBD
*Adolescent-Treated Females*				
PND29	13.93 ± *0.21*	14.04 ± *0.35*	13.98 ± *0.44*	14.36 ± *0.33*
PND45	16.55 ± *0.56*	16.04 ± *0.36*	16.25 ± *0.62*	16.92 ± *0.34*
PND73	18.85 ± *0.45*	18.54 ± *0.53*	18.54 ± *0.70*	19.69 ± *0.34*
*Adult-Treated Females*				
PND64	18.71 ± *0.33*	18.52 ± *0.56*	19.00 ± *0.44*	18.89 ± *0.25*
PND80	20.34 ± *0.34*	19.49 ± *0.50*	20.34 ± *0.36*	19.69 ± *0.15*
PND108	21.31 ± *0.31*	21.10 ± *0.52*	21.43 ± *0.32*	20.87 ± *0.17*
*Adolescent-Treated Males*				
PND29	15.42 ± *0.65*	15.28 ± *0.52*	15.29 ± *0.54*	15.66 ± *0.89*
PND45	20.69 ± *0.29*	19.53 ± *0.49*	20.48 ± *0.55*	19.20 ± *0.63*
PND73	24.61 ± *0.45*	23.85 ± *0.59*	23.88 ± *0.35*	23.09 ± *0.43*
*Adult-Treated Males*				
PND64	24.23 ± *0.53*	23.53 ± *0.48*	25.35 ± *0.50*	24.47 ± *0.51*
PND80	26.41 ± *0.70*	24.83 ± *0.52*	27.61 ± *0.63*	26.24 ± *0.58*
PND108	27.92 ± *0.61*	26.56 ± *0.57*	27.23 ± *1.19*	27.77 ± *0.61*


#### Novel Object Recognition

**FIGURE 6:**
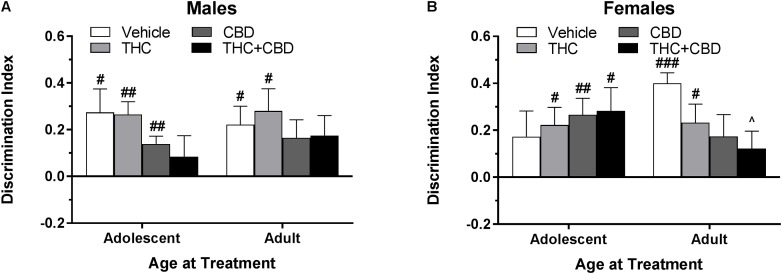
Depicts discrimination of a novel object in the NOR task in pair-housed adolescent and adult males **(A)** and females **(B)** following a history of repeated cannabinoid treatment. Higher positive values indicate more time spent with the novel object. Hashtag indicates significant object discrimination (different from 0) at ^#^*p* < 0.05, ^##^*p* < 0.01, and ^###^*p* < 0.001. Carrot (^∧^) indicates significantly different than respective control at *p* < 0.05, *n*’s = 9–10.

In pair-housed males, one-sample *t*-tests indicated that significant novel object discrimination occurred in mice with an adolescent and adult history of vehicle or THC, as well as mice with an adolescent history of CBD (*p*’s < 0.05). Although some cannabinoid treated groups failed to demonstrate NOR, a Drug^∗^Age at Treatment ANOVA revealed no significant main effects of or interaction of the variables on discrimination index (*p*’s > 0.05). Further, there were no effects of drug history within either age group (*p*’s > 0.05) (Figure 6A), and time spent investigating the objects during the training and test phases were not altered by drug history (data not shown). Discrimination index was not significantly correlated with training investigation time within any drug group (*p*’s > 0.05) (data not shown).

In pair-housed females, mice treated during adolescence with THC, CBD, and THC+CBD as well as mice treated during adulthood with vehicle and THC demonstrated object discrimination to varying levels of significance (*p*’s < 0.05). A Drug^∗^Age at Treatment ANOVA revealed no significant main effects or interaction on discrimination index (*p*’s > 0.05). One-way ANOVAs for each age group indicated a weak trend of drug history in adult-treated animals [*F*(3,33) = 2.46, *p* = 0.08], with a THC+CBD history significantly reducing object discrimination compared to a vehicle history [*t*(33) = 2.56, *p* < 0.05, *d* = 1.51] (Figure 6B). Contrary to the male data, various investigative behaviors in the NOR task were altered by a history of cannabinoids. Females with an adolescent history of CBD spent more time investigating the objects during the training [*t*(35) = 3.32, *p* < 0.01] phase (data not shown). Discrimination index was significantly positively correlated with training investigation in mice treated during adulthood with THC [*r*(9) = 0.740] and THC+CBD [*r*(9) = 0.726] (*p*’s < 0.05) (data not shown), indicating that adult females with a history of THC may require more time to create a detailed memory of the object during the training phase.

#### Elevated Plus Maze Activity

**FIGURE 7:**
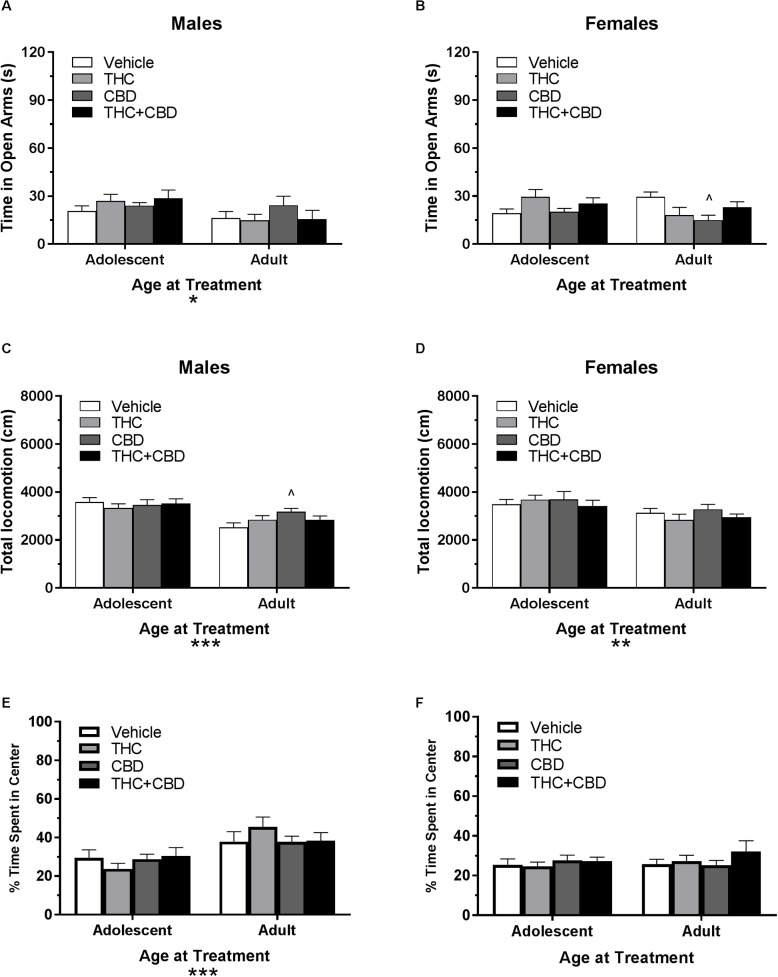
Depicts the effects of repeated cannabinoid treatment in pair-housed adolescent and adult mice on time in the open arms of the EPM (males, **A**; females **B**), total locomotion in the open field (males, **C**; females, **D**), and percent of time spent in the center of the open field (males, **E**; females, **F**). Asterisks indicate a significant main effect at ^∗^*p* < 0.05, ^∗∗^*p* < 0.01, and ^∗∗∗^*p* < 0.001. Carrot (^∧^) indicate significantly different from respective control at *p* < 0.05, *n*’s = 9–10.

In pair-housed males, no significant interaction of Dose^∗^Age at Treatment or effect of drug history was revealed on time spent in the open arms or number of open arm entries (*p*’s > 0.05). There was a main effect of age on both variables (*p*’s < 0.05), with adolescent-treated mice spending more time in the open arms (Figure 7A) and making more open arm entries (data not shown).

In pair-housed females, a Dose^∗^Age at Treatment ANOVA revealed no significant effect of drug history or age at treatment on time spent in the open arms (*p*’s > 0.05). However, there was a significant interaction effect [*F*(3,69) = 3.36, *p* < 0.05]. One-way ANOVAs assessing drug treatment for each group revealed no effect in adolescent-treated mice (*p* > 0.05), but that treatment with CBD during adulthood reduced time in the open arms compared to vehicle [*t*(33) = 2.87, *p* < 0.05, *d* = 1.55] (Figure 7B). There was no significant interaction of Drug^∗^Age at Treatment or effect of drug treatment on number of open arm entries (*p*’s > 0.05). However, there was a significant main effect of age [*F*(1,69) = 5.17, *p* < 0.05], with adolescent-treated mice making more entries (data not shown).

#### Open Field Activity

In pair-housed males, a Drug^∗^Age at Treatment ANOVA revealed no significant interaction or effect of previous drug treatment on total locomotion in the open field (*p*’s > 0.05). There was a significant effect of age [*F*(1,72) = 23.8, *p* < 0.001], with mice treated in adolescence traveling a greater distance. One-way ANOVAs indicated no significant effect of prior drug history in adolescent-treated mice, but a trend toward an effect in adult-treated mice (*p* = 0.072). Mice treated in adulthood with CBD traveled significantly more distance than their vehicle counterparts did [*t*(36) = 2.76, *p* < 0.05, *d* = 1.16] (Figure 7C). Anxiety-like activity in the open field was quantified using percent of time spent in the center of the open field. A Drug^∗^Age at Treatment ANOVA revealed no significant interaction or main effect of drug treatment on anxiety-like activity following a history of cannabinoid administration (*p*’s > 0.05). There was a significant effect of age at treatment [*F*(1,72) = 16.96, *p* < 0.001], with adult-treated mice spending more time in the center of the open field. One-way ANOVAs indicated no significant effect of drug in adolescent- or adult-treated mice (*p*’s > 0.05) (Figure 7E).

In pair-housed females, a Drug^∗^Age at Treatment ANOVA revealed no significant interaction or main effect of drug history on total locomotion in the open field (*p*’s > 0.05). There was a significant main effect of age at treatment [*F*(1,71) = 6.59, *p* < 0.05], with adolescent-treated mice traveling a greater distance. One-way ANOVAs did not indicate a significant effect of drug history for either age group (*p*’s > 0.05) (Figure 7D). A Drug^∗^Age at Treatment ANOVA revealed no significant interaction or main effects on anxiety-like activity following a history of cannabinoid administration (*p*’s > 0.05). One-way ANOVAs indicated no significant effect of drug in adolescent- or adult-treated mice (*p*’s > 0.05) (Figure 7F).

### Hippocampal Western Blot

#### CB1R Protein Expression

A Sex^∗^Age at Treatment ANOVA revealed no significant interaction or main effect of age at treatment on CB1R, IL-1R1, or 5HT1a expression levels in vehicle groups (*p*’s > 0.05). There were main effects of sex, with males vehicle-exposed having greater CB1R [*F*(1,33) = 8.53, *p* < 0.01] and IL-1R1 [*F*(1,35) = 5.76, *p* < 0.05] expression levels than females. However, vehicle-exposed females had greater 5HT1a expression [*F*(1,33) = 43.68, *p* < 0.001] than males.

Drug^∗^Age at Treatment ANOVAs revealed no significant interaction or main effect of drug or age at treatment on CB1R, IL-1R1, or 5HT1a expression levels in males or females (*p*’s < 0.05). Further, one-way ANOVAs within each age group and sex revealed that no drug history group was significantly different from their vehicle counterpart (data not shown).

## Discussion

The current project comprises a set of experiments examining age- and sex-effects of cannabinoid administration on acute and long-term behaviors. Although many significant acute actions of cannabinoids were demonstrated, there were minimal long-term effects associated with a history of repeated drug administration across age and sex. However, the significant effects reported are robust. The effect sizes reported herein indicate that significantly different *p*-values represent group differences wherein at least 73% of the treatment group is beyond the mean of the control group (Magnusson, 2014).

### Experiment 1: THC and CBD Dose Responses

Based on previous studies (e.g., Guimarães et al., 1990; Onaivi et al., 1990; Kasten et al., 2017), acute THC and CBD were expected to have respective anxiogenic and anxiolytic effects in each age group. Although THC produced a strong anxiogenic effect in adults of both sexes, CBD did not produce an anxiolytic effect (Figure 2, 3). The anxiogenic effect was milder in adolescents, with only significant effects seen in the open field metric. However, adolescent control mice demonstrated more anxiogenic activity in the EPM than adults, suggesting that a higher dose of THC may be required to significantly increase anxiety-like behavior in this assay. The 10 mg/kg dose of THC produced a decrease in locomotor activity in all mice, including an insignificant decrease of 31.8% in adolescent males, suggesting that it was pharmacologically active in all groups. Significant reductions in locomotor activity were only correlated with anxiety-like activity in adult males that received 5 mg/kg THC. Interestingly, this group did not show significant changes in anxiety-like activity versus their vehicle counterparts. However, there were no significant correlations between locomotor activity and the increases in anxiety-like activity seen in mice administered 10 mg/kg THC (Figure 2). This indicates that, in this study, activity levels did not directly contribute to changes in anxiety-like activity.

### Effects of Cannabinoids on Anxiety-Like and Locomotive Behavior

Although the acute effects of THC and CBD were tested in Experiment 1 to inform drug doses for Experiments 2 and 3, mice in Experiment 2 were also tested for acute effects of cannabinoids on EPM and open field activity to include the combination of THC+CBD (Figure 5). Notably, the only significant anxiogenic response on the EPM in Experiment 2 was in adult males administered THC+CBD. This is in opposition to Experiment 1, in which THC alone produced an anxiogenic profile (Figure 2). However, the anxiogenic profile of THC was still present in adult males and females in the open field (Figure 5).

Two major differences exist between procedures in Experiments 1 and 2. Mice were single-housed in Experiment 1 as in our previous work (Kasten et al., 2017), but pair-housing was used in Experiment 2 to avoid potential confounds of long-term isolation housing on EPM activity and object recognition (Hueston et al., 2017; Lin et al., 2018). Secondly, Experiment 1 only observed activity in the EPM and open field following one drug injection. In Experiment 2, EPM and open field followed the NOR task. Therefore, mice received their second drug administration prior to EPM and open field in Experiment 2 (Table 2). Development of rapid tolerance to cannabinoids and/or influence of housing may have contributed to these differential findings. It has been previously demonstrated that strong anxiogenic responses in mice on the EPM following an initial acute injection are no longer present on the 5th day of injections (Onaivi et al., 1990). However, our previous work has demonstrated that single-housed adult male mice have a persistent anxiogenic response in the EPM following a second injection with 10 mg/kg THC (Kasten et al., 2017). Although short-term isolation housing in B6 mice does not alter activity in the EPM alone (Lin et al., 2018), the combination of isolation housing with injection stress and drug exposure may potentiate anxiety-like activity. Examining EPM behavior following the first injection of THC+CBD may have revealed an anxiogenic response in more groups, and this response may have been synergistically greater than THC alone, similar to the center field activity in adult females. The percent of time spent in the center of the open field results more closely resemble the anxiogenic effect in adults on the EPM following one dose of THC (Figure 2). While it is tempting to assert that anxiogenic activity should be consistent between the EPM and center metrics in the open field, a recent meta-analysis by Mohammad et al. (2016) indicates that these two tasks do not reliably reproduce one another, and should not be interpreted as reflecting the same behavioral motivation.

Although the anxiogenic effects of THC in the EPM were attenuated following a second THC administration, locomotor effects persisted (Figure 5). THC reduced total locomotion in all groups, whereas THC+CBD reduced total locomotion in all groups but adolescent females. Similar to the dose-response results, overall changes in activity levels had a tenuous relationship with anxiety-like activity. Although reductions in activity were correlated with increased anxiety-like activity under some instances, this relationship was only present in males for percent of time spent in the center of the open field, but not open arm entries in the EPM. As adolescent males did not demonstrate a locomotor depressant effect following one dose of THC (Figure 2), this may indicate that locomotor depression may develop over repeated THC injections. These results, paired with those of the EPM, may indicate both tolerance and increased sensitivity (behavioral sensitization) to repeated THC injections in different behavioral assays in the same mice. Support for these opposing processes has been previously reviewed by Pertwee (2008). Due to the regional differences in density, location, and coupling efficiencies of CBRs, repeated cannabinoid administration may reduce CB1R density and coupling efficiency at a different rate across brain regions. Therefore, rapid tolerance may develop for some, but not all *in vivo* effects of cannabinoids (Pertwee, 2008).

Long-lasting effects of cannabinoid exposure were minimal. One primary concern is that these tests may be susceptible to one-trial tolerance, which is particularly notable in the EPM (Walf and Frye, 2007). Although there was a 6-week period between the acute and long-term tasks, the time spent in the open arms of the EPM in the vehicle groups at the long-term time point was approximately 1/3^rd^ of their open-arm time at the acute time-point, potentially contributing to a floor effect. Total locomotion in the open field and percent of time spent in the center of the open field were relatively resilient to effects of repeated exposures (Figure 5, 7). Interestingly, an adult history of CBD resulted in anxiogenic activity in females, whereas an adolescent history of THC in females increased the number of open arm entries. However, this change in arm entries did not translate to more time spent in the open arms (Figure 7). Our previous work in single-housed mice also demonstrated minimal long-lasting effects of THC exposure, only finding that repeated exposure in males during adulthood lead to significantly greater percentage of total locomotion in the center of the open field (Kasten et al., 2017). This anxiolytic phenotype is in direct opposition to the anxiogenic phenotype demonstrated by Demuyser et al. (2016) using B6 mice from Charles River France that were pair-housed further into adulthood. As a whole, current work in the field suggests a tenuous relationship between cannabinoids, anxiety, and single-housing (Võikar et al., 2005; Lopez and Laber, 2015; Demuyser et al., 2016; Kasten et al., 2017; Lin et al., 2018). However, the disparate findings in the current study highlight the importance of standardizing housing conditions across studies.

### Effects of Cannabinoids on Object Recognition Memory

Previous studies using a range of THC doses have demonstrated an acute effect on object recognition memory in CD-1 mice (Barbieri et al., 2016; Busquets-Garcia et al., 2018), but not other rodent strains (Ciccocioppo et al., 2002; Long et al., 2010; Swartzwelder et al., 2012; Kasten et al., 2017). Acute effects of CBD or THC+CBD have not been reported. As hypothesized, all mice but adolescent males significantly discriminated the novel object when injected with vehicle post-training (Figure 4). These objects were specifically chosen for their ability to produce significant discrimination under naïve conditions (Figure 1), suggesting that a single injection produces similar deficits in object recognition as restraint stress following the object training session in adolescent males (Kim et al., 2018). Interestingly, THC administration trended toward rescuing the injection effect in adolescent male mice and the CBD group also significantly discriminated, whereas adult male mice only showed significant object discrimination following the vehicle injection (Figure 4A). Females did not display a similar stark age-effect of injection or cannabinoid action as the males (Figure 4B). A shorter inter-trial interval may have produced more consistent and significant acute cannabinoid effects in the NOR task (Barbieri et al., 2016; Busquets-Garcia et al., 2018). Although it has been suggested that more time spent with the objects during training may indicate better performance in the test session (Cohen and Stackman, 2015), we found no consistent evidence supporting this relationship when acute cannabinoids were administered following the training session.

The effects of cannabinoid history were tested 23 days following the last of eight injections. Based on prior research in rats and mice, it was hypothesized that mice with an adolescent history of THC would show impaired object recognition (Quinn et al., 2008; Realini et al., 2011; Zamberletti et al., 2012; Kasten et al., 2017; Kevin et al., 2017; Murphy et al., 2017), and that addition of CBD to THC would rescue this deficit (Fagherazzi et al., 2012; Cadoni et al., 2013; Campos et al., 2015; Gomes et al., 2015). Our hypothesis was not supported. Males and females treated with THC during adolescence significantly discriminated the novel object following a period of drug removal (Figure 6). Although six injections over the same age period were sufficient to impair object recognition memory in our previous study (Kasten et al., 2017), the use of pair housing may reduce susceptibility to THC’s impairing effects (Võikar et al., 2005). A shorter inter-trial interval between training and testing or a more frequent or increasing dosing regimen over the same age period may have produced the previously seen deficits, such as the every-day dosing paradigm over at least 10 days (Quinn et al., 2008; Realini et al., 2011; Zamberletti et al., 2012; Murphy et al., 2017; Rodríguez et al., 2017).

A few studies have used adult controls to observe whether the effects of THC treatment on object recognition memory are specific to adolescent administration. O’Tuathaigh found no effect of THC history at either age, Quinn et al. (2008) and Murphy et al. (2017) found no effect of adult THC treatment on later object recognition memory, whereas our previous findings demonstrated that an adult history of THC rescued a significant impairment in object recognition memory seen in vehicle-treated male mice (Kasten et al., 2017). However, the current study found no major differences between treatment groups in adult-treated males (Figure 6A). Conversely, the adult-treated females showed a step-wise response to cannabinoid treatment, with the vehicle group showing very strong object discrimination (Figure 6B). The females that received THC+CBD during adulthood demonstrated significantly impaired object discrimination compared to the vehicle group. Further, the THC and THC+CBD adult-treated females had training investigation times that were significantly positively correlated with discrimination index, indicating that increased exploration during training facilitated object recognition memory in the test session and that previous THC exposure in this group may require more cognitive effort to successfully complete a task. This interpretation is supported by findings in the human visual paired-comparison task, which indicate that impaired visual recognition in high-risk infants can be bolstered by increasing the length of time to familiarize with an object (Burbacher and Grant, 2012).

### Western Blots

The current study used Western blotting to identify protein expression of CB1R, IL-1R1, and 5HT1a following cannabinoid history in the hippocampus. Although the hippocampus is necessary for the current NOR design (Cohen and Stackman, 2015) and shows high levels of THC metabolism (Leishman et al., 2018), no significant effects were found in protein levels when examining homogenized whole hippocampal tissue. Due to the changes in CB1R expression over development (Rodríguez de Fonseca et al., 1993; Romero et al., 1997; Verdurand et al., 2011; Lee and Gorzalka, 2012) paired with changes in density following repeated cannabinoid administration (Pertwee, 2008), the lack of persistent change in CB1R expression levels was surprising. However, samples were taken approximately a month following the final cannabinoid treatment. Samples gathered closer to the completion of cannabinoid injections may have revealed changes in protein expression that begin to normalize at 1 month post-treatment. Further, overall protein expression may not consistently reflect changes seen regionally within the hippocampus, at the cellular level (synaptic versus extrasynaptic, receptor internalization), or functional changes in existing receptors.

5HT1aR and IL1-R1 were chosen as secondary targets due to the ability of CBD to influence behavior via these receptors (e.g., Russo et al., 2005; Campos et al., 2012) and the role of inflammatory shifts and interleukin-1 in hippocampal-dependent memory (Goshen et al., 2007; Hueston et al., 2017). Although no changes were found in western blot levels, the role of 5HT1a receptors in the NOR-impairment seen in adult-treated females is of particular interest due to the relationship between changes in estrogen and the 5HT1a receptor system that result from repeated stress exposure, such as chronic injections. Stressors reduce estrogen release in fully developed females, potentially resulting in a shift toward more hetero- and less post-synaptic 5HT1a receptors being expressed at raphe nucleus → hippocampal synapses (Toufexis et al., 2014). Increased heteroreceptor activation at the raphe nucleus results in suppression of serotonin transmission (Glikmann-Johnston et al., 2015), which is critical for object recognition (Busquets-Garcia et al., 2016). The combination of THC with CBD may increase the time of action of CBD at 5HT1a receptors (Stout and Cimino, 2014), resulting in long-term impairment of hippocampal memory development in sexually developed females that were administered THC+CBD. Conversely, the adolescent brain may be undergoing rapid developments in this system, which makes it less susceptible to long-term consequences of repeated exposure. The role of 5HT1a receptors in this phenomenon could be investigated using pharmacological or neurochemical approaches including WAY-100,135 co-administration, conditional receptor knockdown, electrophysiology, and *in situ* hybridization.

## Conclusion

The current studies examined age- and sex-effects of cannabinoid administration on acute and long-term behaviors. Although many significant acute actions of cannabinoids were observed, there were minimal long-term effects associated with repeated drug administration across age and sex. Contrary to our initial hypotheses, acute administration of THC+CBD resulted in behavioral deficits, potentially due to the ability of administration of two or more cannabinoids to prolong metabolism and drug availability (Klein et al., 2011; Stout and Cimino, 2014; Murphy et al., 2017). THC+CBD administration also resulted in long-lasting effect of cannabinoids, wherein females repeatedly treated in adulthood demonstrated impaired object recognition memory. Although CBD is generally considered to be a safe, non-intoxicating therapeutic (e.g., Leweke et al., 2012; Englund et al., 2013, 2017), recent studies in humans have indicated that CBD alone may produce intoxicating effects and enhance psychotic symptoms dependent upon individual cannabinoid history (Morgan et al., 2018; Solowij et al., 2019). The current results indicate that females may have a different sensitivity to CBD, potentially due to its actions at 5HT1a receptors. In females, stress, hormones, and 5HT1a activation may be more likely to contribute to negative outcomes of cannabinoid usage, such as impaired cognition or increases in susceptibility for major depression (see Grigoriadis and Robinson, 2007; Martin et al., 2009).

The findings that THC+CBD resulted in increased impairment were in conflict with the hypotheses that combining THC+CBD would result in reduced impairment. Concerning medical and recreational use, this may indicate that higher concentrations of CBD with lower concentrations of THC serve to extend moderate and beneficial effects of THC administration. However, at a higher ratio, such as the 1:2 ratio used in the current studies, CBD may enhance and prolong the negative effects of THC use. A range of THC:CBD ratios, including the commercially popular 2:1 ratio or the medically popular 1:1 ratio (Jikomes and Zoorob, 2018), should be investigated to fully understand how their pharmacological interaction affects behavior.

There were minimal long-lasting effects of cannabinoid injections, suggesting that both male and female mice demonstrate a relative robustness against cannabinoid use at both adolescent and adult time points. This study alone may indicate that cannabinoids are more suitable for long-term medical treatment and may be more appropriate as an intervention for diseases that occur during childhood. However, only eight injections were given in the current study, and the adolescent treatment regimen ended at PND45. PND45 is roughly equivalent to 18 years of age in humans (Lee and Gorzalka, 2012), which is the same period of age when self-reports of past-month cannabis use nearly triples (Azofeifa et al., 2016). Previous studies using escalating THC doses over the same age period in adolescent rats have demonstrated long-term deficits in object recognition, indicating that the dosing regimen may also play a large role in these findings.

The choice of behaviors used in the current studies must be considered. A recent review by the National Academies of Sciences (2017) reported that there is moderate evidence of cognitive impairment following acute cannabinoid use and limited evidence of long-lasting cognitive impairment following abstinence. There is also limited evidence of a relationship between development of non-social anxiety disorders and cannabis use, although anxiety-like and sedative responses should be monitored. Although the current behaviors were chosen based on previous literature and findings in our own lab which suggested that cannabinoid treatment results in deficits in object recognition memory and unconditioned anxiety, it is possible that the role cannabinoid use plays in these impairments is more limited than initially expected. The use of preclinical behavioral assays that are analogs to the conditions that the National Academies of Sciences have more strongly associated with cannabinoid use - such as development of other substance use disorders, social anxiety, depressive symptomology, and psychoses – may reveal more effects than the behavioral assays chosen herein. Therefore, the current studies may not represent the trajectory of behavioral outcomes following actual medical or recreational cannabinoid usage.

## Author Contributions

CK and SB were responsible for the study concept, design, and interpretation of the findings. CK and YZ were responsible for acquisition and analysis of the data. CK was responsible for the first draft of the manuscript. CK, YZ, and SB contributed to critical review and approval of the version for publication.

## Conflict of Interest Statement

The authors declare that the research was conducted in the absence of any commercial or financial relationships that could be construed as a potential conflict of interest.
